# Immunoglobulins against the surface of *Plasmodium falciparum-*infected erythrocytes increase one month after delivery

**DOI:** 10.1186/1475-2875-11-130

**Published:** 2012-04-25

**Authors:** Alfredo Mayor, Elisa Serra-Casas, Eduard Rovira-Vallbona, Alfons Jiménez, Llorenç Quintó, Betuel Sigaúque, Carlota Dobaño, Azucena Bardají, Pedro L Alonso, Clara Menéndez

**Affiliations:** 1 Barcelona Centre for International Health Research, (CRESIB), Hospital Clínic-Universitat de Barcelona, Barcelona, Spain; 2 Centro de Investigação em Saúde de Manhiça (CISM), Maputo, Mozambique; 3 National Institute of Health, Ministry of Health, Maputo, Mozambique

**Keywords:** Malaria, Pregnancy, Postpartum, Antibody responses, *Plasmodium falciparum*

## Abstract

**Background:**

The risk of *Plasmodium falciparum* malaria increases during pregnancy and at early postpartum. Immunological and physiological alterations associated with pregnancy that persist after delivery may contribute to the susceptibility to *P. falciparum* during early postpartum period.

**Methods:**

To determine changes in antibody-mediated responses after pregnancy, levels of Immunoglobulin G (IgGs) specific for *P. falciparum* were compared in 200 pairs of plasmas collected from Mozambican women at delivery and during the first two months postpartum. IgGs against the surface of erythrocytes infected with a *P. falciparum* chondroitin sulphate A binding line (CS2) and a paediatric isolate (MOZ2) were measured by flow cytometry.

**Results:**

IgG levels against CS2 and MOZ2 were higher at postpartum than at delivery (p = 0.033 and p = 0.045, respectively) in women without *P. falciparum* infection. The analysis stratified by parity and period after delivery showed that this increase was significant in multi-gravid women (p = 0.023 for CS2 and p = 0.054 for MOZ2) and during the second month after delivery (p = 0.018 for CS2 and p = 0.015 for MOZ2).

**Conclusions:**

These results support the view that early postpartum is a period of recovery from physiological or immunological changes associated with pregnancy.

## Background

Infections due to several microbial pathogens and auto-immune diseases have been shown to begin or worsen during early postpartum period [1]. In particular, the risk of *Plasmodium falciparum* malaria increases during pregnancy [2] and has been suggested to remain high at early postpartum compared to the same women during pregnancy [3] and to non-pregnant women [4]. However, other studies have suggested that the rate of parasitaemia decreases after delivery and that women who were parasitaemic at delivery cleared their parasitaemia spontaneously at early postpartum [5,6]. Whereas susceptibility to malaria during pregnancy has been attributed to lack of antibodies able to block binding of *P. falciparum* to chondroitin sulphate A (CSA) in the placenta [7], little is known about the anti-malarial immune responses of women during the first months after delivery.

It has been suggested that immunity is altered during pregnancy to promote tolerance to foetal antigens [8]. Maintenance of an essentially type 2 cytokine environment, modulation of lymphocyte responses and redistribution of regulatory T cells (reviewed in [1]) appear to be essential for a successful pregnancy. It has been speculated that the period of recovery from immunological and physiological alterations associated with pregnancy may still render puerperal women susceptible to malaria [3].

There is lack of information on the dynamics of antibodies against *P. falciparum* during early postpartum [9-11]. The aim of the present study was to determine changes in the level of antibodies against *P. falciparum* during the first two months postpartum that may suggest alterations of humoral immunity during pregnancy [1]. To address this, immunoglobulin G (IgG) levels against the surface of *P. falciparum*-infected erythrocytes were compared in paired samples collected at delivery and postpartum of the same women, taking into consideration the effect of placental infection, parity, HIV infection and postpartum period.

## Methods

### Study area and population

The study was conducted at the Centro de Investigação em Saúde de Manhiça (CISM) in Manhiça District, southern Mozambique, between 2003 and 2005. Malaria transmission is perennial with some seasonality [12]. During the study, intermittent preventive treatment during pregnancy (IPTp) with sulphadoxine-pyrimethamine (SP) was not yet recommended by the Ministry of Health and malaria control in pregnancy relied exclusively upon case management.

### Study design

This study was conducted in the context of a randomized, double blind, placebo-controlled trial of IPTp with SP (trial registration number: NCT00209781) [13]. Women enrolled into the study received a long-lasting insecticide-treated net and were randomized to receive two placebo or SP doses from the second trimester, at least one month apart. At the time of delivery and four to eight weeks later, thin and thick smears were prepared and examined for malarial parasites according to quality-control procedures [14]. Peripheral blood was collected into EDTA vacutainers, centrifuged and plasma was stored at −20°C. Blood samples from the placenta and periphery of pregnant women at delivery, as well as at postpartum, were collected onto filter papers (Schleicher & Schuell number 903TM). Placental biopsies from the maternal side of the placenta were collected for histological examination [13]. The last 200 women recruited in the main trial (n = 1030) with all demographic data and available plasma and filter papers from both delivery and postpartum were included in this study.

Written informed consent was obtained from all study participants. Malaria clinical episodes were treated following national guidelines at the time of the study. The study was approved by the national Mozambican ethics review committee and the Hospital Clínic of Barcelona ethics review committee.

### *Plasmodium falciparum* detection by PCR

DNA was extracted from a blood drop of 50 μl onto filter paper with an ABIPrism 6700 automated nucleic acid work station (Applied Biosystems) and finally re-suspended in 200 μl water. Five μl of sample were screened for *P. falciparum* DNA by real-time quantitative PCR (qPCR) [15].

### Measurement of antibody responses against the surface of infected erythrocytes

The chondrointin sulphate A (CSA)-binding strain CS2 [16] (MRA-96, MR4, ATCC®, Manassas, VA) and a Mozambican paediatric non-CSA-binding isolate (MOZ2) [15] were cultured under standard conditions, synchronized at ring stage and cryopreserved in multiple aliquots at a parasitaemia of 1–3 %, until used for antibody determinations. Matched plasma samples from women at delivery and postpartum were tested in the same experiment for recognition of CS2 and MOZ2 by flow cytometry, as previously described [15]. To minimize inter-assay variations, all the experiments were conducted with different aliquots of the same batch of cryoperserved ring-stage parasites. Parasites were thawed, matured to trophozoite and re-suspended at 1 % haematocrit. The suspensions of infected erythrocytes were sequentially incubated with test plasma at 1:20 dilution, polyclonal rabbit anti–human IgG (DakoCytomation; dilution 1:200) and AlexaFluor donkey anti–rabbit IgG (Invitrogen; dilution 1:1000) plus 10 μg/mL of ethidium bromide. Data from 1000 events in the channel for ethidium bromide–labelled erythrocytes were acquired with a Becton-Dickinson FACSCalibur flow cytometer. Reactivity against the surface of infected erythrocytes was expressed as the difference between the mean fluorescence intensity (MFI) of infected erythrocytes and the MFI of uninfected erythrocytes.

### Definitions and statistical methods

Peripheral infection at delivery and postpartum was defined as the presence of parasite DNA detected by qPCR. Placental infection was defined as the presence of parasites detected by qPCR or the presence of only pigment observed by histology (past infection). Women were classified as primigravidae (PG) if they were pregnant for the first time, secundigravidae (SG) if they were in their second pregnancy and multigravidae (MG) if they reported at least two previous pregnancies. Postpartum periods were defined as early if the blood sample was collected during the first 30 days after delivery, or late if the sample was collected during the second month post-delivery. Proportions and continuous variables between independent groups were compared by the Fisher´s exact and Mann Whitney’s test, respectively. Wilcoxon rank sum test was used for pair-wise comparisons of levels of IgGs (MFI values) in samples at delivery and postpartum from the same women. A p-value <0.05 was considered statistically significant. Data analysis was performed using Stata 11 (Stata Corporation, College Station, TX, USA).

## Results

### Characteristics of the study women

Among the 200 women included in the study, 50 (25 %) were PG, 37 (19 %) SG, and 113 (56 %) MG; median age was 23 years (interquartile range [IQR] 19–28); 50 women (25 %) were HIV positive and 100 (50 %) had received IPTp with SP. Overall, they were comparable with the 1,030 total women participating in the randomized trial [13] in terms of IPTp intervention, parity and age (all p-values > 0.306). By microscopy, 16 (8 %%) women were infected in their peripheral blood at delivery and eight (4 %) at postpartum. qPCR detected infections in 48 (24 %) placental blood samples, 55 (27 %) peripheral samples and 20 (10 %) postpartum samples. Thirty-seven (19 %) women were infected at delivery in both peripheral blood and placenta, 18 (9 %) only in peripheral blood and 11 (6 %) only in the placenta. Histological examination of the placental sections showed the presence of 24 (12 %) *P. falciparum* active infections (13 with only parasites and 11 with both parasites and haemozoin) and 68 (34 %) past infections Among these 24 placentas with active infection, six were negative by qPCR (five acute and one chronic infection). Histological examination of the 152 placentas that were negative by qPCR showed that 93 were negative both for parasite and pigment, 53 had pigment, five only parasites and one both parasites and pigment. Overall, there was no evidence of parasite by qPCR nor pigment by histology in 98 (49 %) of the placentas.

Among the 200 postpartum samples, 39 (19 %) were collected during the first month after delivery (early postpartum) and 161 (81 %) during the second month post-delivery (late postpartum). Proportion of MG was similar among early (21/36 [58 %]) and late postpartum women (80/144 [56 %]; p = 0.852) and among HIV negative (84/150 [56 %]) and positive women (29/50 [58 %]; p = 0.302).

### Effect of *Plasmodium falciparum* infection at postpartum on IgG levels

Infection at postpartum was associated with higher levels of IgGs against CS2 and MOZ2, compared to levels in women without infection (Table 1). As infection during postpartum is highly associated with infection at delivery [17], and to check for the possibility that higher levels of IgGs after delivery may be due to the boosting of antibody responses by placental infection [18], IgG levels by postpartum infection were compared among women with no detectable active or past placental infection (n = 98). In the absence of placental infection, no association was found between postpartum infection and increased level of IgGs against the surface of IEs (Table 1).

**Table 1:** IgG levels against the surface of erythrocytes infected with the parasite lines CS2 and MOZ2, by infection at postpartum

		**Postpartum -**		**Postpartum +**		
		**Median IQR**		**Median IQR**		**p**
**All women (n = 200)**						
	**CS2**	78.9	28.4-305.9	526.9	163.4-766.1	**<0.001**
	**MOZ2**	45.8	23.8-83.6	83.3	49.2-127.6	**0.007**
Women without placental infection (n = 98)*						
	**CS2**	47.4	22.4-184.5	53.3	39.2-104.5	0.845
	**MOZ2**	36.4	19.3-62.4	44.6	17.6-52.0	0.991

IQR: Inter-quartile range.

* Analysis excluding women with placental malaria to rule out the boosting of antibodies by infection at delivery.

### IgG levels at delivery and postpartum

Levels of IgGs specific for CS2 and MOZ2 were higher at postpartum than at delivery in 115 (58 %) and 120 (60 %) of the 200 women, respectively (Table 2). The paired analysis showed that levels of IgGs against MOZ2 increased at postpartum compared to delivery (p = 0.001), and a similar trend was found for IgGs against CS2 (p = 0.054) (Table 2). Analysis stratified by parity, postpartum period, HIV status and intervention showed that this difference was significant in MG women (CS2 and MOZ2), at late postpartum (MOZ2), in women who received IPTp (CS2 and MOZ2; differences for MOZ2 were also observed among women who received placebo), and in HIV-negative women (MOZ2; Table 2). To check for the possibility that increased levels of antibodies at postpartum might be due to the boosting effect of *P. falciparum* infections either in the placenta or at postpartum, IgG levels among women without evidence of infection (n = 95) were also compared between delivery and postpartum (Table 2). This analysis confirmed that postpartum was associated with higher levels of antibodies against both parasite lines among women without evidences of placental infection during pregnancy. The analysis stratified by parity and postpartum period showed differences in MG women (statistically significant for CS2 and a trend for MOZ2) and at late postpartum (Table 2). No consistent pattern was found for HIV infection, since IgG levels were higher at postpartum than at delivery both among HIV-negative (CS2) and among HIV-infected women (MOZ2; Table 2).

**Table 2:** Number of women with a positive or negative difference in IgG levels between postpartum and delivery, and magnitude of the difference

			**All (n = 200)**				**Placenta and postpartum negative (n = 95)**			
			**n**_**>0**_	**n**_**<0**_	**Difference**	**p**^**a**^	**n**_**>0**_	**n**_**<0**_	**Difference**	**p**^**a**^
					**(%)**				**(%)**	
**All women**										
		**CS2**	115	85	5.8	0.054	57	38	6.1	**0.033**
		**MOZ2**	120	80	9.8	**0.001**	56	39	10.0	**0.045**
**By parity**										
	**Primigravidae**									
		**CS2**	24	26	4.7	0.828	9	9	−1.4	0.557
		**MOZ2**	31	19	8.3	0.121	12	6	21.3	0.349
	**Secundigravidae**									
		**CS2**	20	17	0.8	0.886	9	7	3.5	0.351
		**MOZ2**	21	16	6.7	0.213	7	9	−0.1	0.569
	**Multigravidae**									
		**CS2**	71	42	13.9	**0.015**	39	22	18.4	**0.023**
		**MOZ2**	68	45	10.4	**0.009**	37	24	10.0	0.054
**By postpartum period**										
	**Early**									
		**CS2**	24	15	19.1	0.116	9	10	−3.5	0.904
		**MOZ2**	22	17	17.7	0.357	8	11	−0.1	0.658
	**Late**									
		**CS2**	91	70	3.3	0.197	48	28	6.9	**0.018**
		**MOZ2**	98	63	8.8	**0.002**	48	28	12.4	**0.015**
**By IPTp**										
	**Placebo**									
		**CS2**	57	43	7.5	0.511	27	19	9.0	0.145
		**MOZ2**	58	42	10.2	**0.029**	25	21	8.8	0.195
	**SP**									
		**CS2**	58	42	4.1	**0.047**	30	19	3.6	0.106
		**MOZ2**	62	38	9.2	**0.015**	31	18	10.0	0.130
**By HIV status**										
	**Negative**									
		**CS2**	90	60	8.5	0.101	48	29	7.6	**0.029**
		**MOZ2**	88	62	7.6	**0.008**	44	33	10.0	0.204
	**Positive**									
		**CS2**	25	25	−1.1	0.527	9	9	−1.1	0.879
		**MOZ2**	32	18	16.7	0.069	12	6	15.9	**0.035**

^a^ Wilcoxon rank sum tests for pair-wise comparisons between MFIs for CS2 and MOZ2 at delivery and postpartum.

n_>0_ indicates the number of women with IgG levels higher at postpartum than at delivery and n_<0_ the number of women with IgG levels lower at postpartum than at delivery.

Difference (%) denotes the median difference in IgG levels between postpartum and delivery samples expressed in % of levels at delivery.

PG, primigravidae; SG, secundigravidae; MG, multigravidae; IPTp, intermittent preventive treatment in pregnancy; SP, sulphadoxine-pyrimethamine.

Analysis including all women and only those without infection were stratified by parity, postpartum period, HIV status and IPTp administration.

## Discussion

The importance of pregnancy-associated malaria does not end at delivery, as the postpartum period is suggested to be also a time of additional vulnerability to *P. falciparum*[3,4]. However, the causes of increased susceptibility to malaria at postpartum remain to be explained. This study shows that antibody responses against the surface of *P. falciparum*-infected erythrocytes are higher at postpartum than in the same women at delivery. This increase was observed both for IgGs against a paediatric isolate and a CSA-binding line, suggesting this is a general phenomenon not-specifically related to placental parasites.

These results are not contradictory to previous reports showing waning of antibodies against CSA-binding parasites six months after delivery [19], as these antibodies are expected to naturally decay with time in absence of exposure to placental-type parasites. Another study conducted among primigravidae Ghanaian women showed that antibody levels against the surface of infected erythrocytes tended to increase from second trimester to six weeks postpartum [11], although this increase was not confirmed in other studies [9,10]. In the same direction, IgGs against Pf155/RESA was shown to increase at postpartum among primigravidae women from Cameroon [20]. The reason for these contradictory results may be owing to differences in the sampling time-points during postpartum, the parity groups included in the study, the prevalence of infection at delivery that boosts antibody responses, as well as the statistical methods used to analyse the data. Also, as previously suggested [20], the regulation of antibody production and the triggering of antibody-secreting cells at delivery and postpartum may depend on the specificity of the antigen.

The increase of antibodies after delivery observed in this study can be partially explained by the boosting of antibodies associated with placental or postpartum infections that may increase IgGs levels postpartum. Women who received IPTp with SP (being at reduced risk of infection during pregnancy because of the intervention and thus expecting a lower boosting of antibodies) had lower antibody levels specific for CS2 at delivery compared to postpartum. However, such a difference was not observed in women who received placebo, suggesting that other factors apart from the boosting of antibodies at delivery might contribute to the increase of antibodies at postpartum. Lower levels of IgGs at delivery might be explained by expansion of plasma volume in pregnant women. However, it has been shown that, similar to observations from the current study, geometric mean titres of antibodies against herpes simplex virus and measles declined during pregnancy and returned to initial values by the end of the postpartum, even after taking into account the effect of haemodilution [21]. These observations, together with other studies showing that several macromolecules in plasma, such as α_2_-macroglubulins, do not change during pregnancy [22], suggest that the contribution of plasma expansion to the increase of antibodies after delivery may be small. Finally, pregnancy-associated immuno-modulation affecting humoral responses might also contribute to the lower level of antibodies at delivery compared to postpartum.

Contradictory results about the effect of pregnancy on the humoral immune system have been reported. Some studies have suggested that the maternal immune system may tolerate foetal antigens by suppressing cell-mediated immunity while retaining normal humoral immunity [8]. However, lower antibody levels against specific infectious agents in pregnant *versus* non-pregnant women have also been described [21,23-25], as well as reductions in other antibodies during pregnancy [26-29]. Results from the current study suggest that pregnancy does not impair acquisition of antibodies against pathogens [30], as shown by the boosting of anti-malarial antibodies associated with *P. falciparum* infection. However, one may speculate that antibody responses may decline during pregnancy in the absence of antigenic stimulation, especially if the activity of the cells involved in the production of antibodies is modulated by pregnancy-associated factors. This latter interpretation may be supported by the observed increase in antibody responses after delivery at late postpartum (i.e. immunity takes some time to recover after delivery) and among MG women (i.e. PG may need more time to restore their basal levels of antibodies).

This study has three important limitations. First, the study focused on the analysis of antibodies against the surface of infected erythrocytes, which are known to have a key role in the development of anti-malarial immunity during pregnancy [7], but limitations of plasma volume did not allow to measure IgGs against other *P. falciparum* antigens or total antibodies. Second, comparisons stratified by parity and postpartum period should be taken with caution due to a reduction of the power of the analysis. And finally, it was not possible to assess the clinical relevance of the increase in antibodies after delivery, given the lack of knowledge on the precise immune mechanisms mediating protection against malaria in pregnancy and the levels needed to achieve this protection.

## Conclusions

Results of this study support the view of early postpartum (i.e. the first month after delivery) as a period in which the level of anti-malarial antibodies gradually increase after delivery, possibly due to recovery from pregnancy-associated physiological and immune changes [1,8]. These re-adjustments might still render women susceptible to new malaria infections and/or allow parasites infecting pregnant women to persist during the early postpartum period [4]. Further studies are needed to understand the mechanisms whereby immunity may recover at postpartum and the implications on resistance to *P. falciparum*.

## Competing interests

The authors have no commercial or other association that may pose a conflict of interest.

## Authors’ contributions

AM conceived the study, performed the statistical analysis and drafted the manuscript; ES and AJ carried out the immunological and molecular determinations; LQ participated in the statistical analysis; ERV, CD and AB participated in the interpretation of results and the drafting of the manuscript; BS participated in the acquisition of data; PLA and CM contributed to the interpretation of the results and the drafting of the manuscript. All authors have read and approved the manuscript.
